# Supplementary material for: Strong plates enhance mantle mixing in early Earth
Source: Nat Commun. 2018 Jul 13;9:2708. doi: 10.1038/s41467-018-05194-5 (PMC6045636; doi:10.1038/s41467-018-05194-5)
Supplement: Supplementary file 1 — Supplementary Information [file 41467_2018_5194_MOESM1_ESM.pdf]

Supplementary information

Strong Plates Enhance Mantle Mixing in Early Earth

Supplementary Table 1 - **List of simulations and results**. The columns show: Simulation - the simulation numbers; Plate age - the initial subducting plate age in Myr; ($\gamma_{rw\text{-}pv}$; $\gamma_{rw\text{-}pv+mw}$) - the Claperon slopes; ΔT_{pot} - the temperature increase, Ra - the thermal Rayleigh number (eq. 4), $Rb_{rw\text{-}pv+mw}$ - the endothermic phase Rayleigh number (eq. 6); P - the phase buoyancy number (eq. 5); t_{670} - the time to reach the 670 km; t_{end} - the time of the end of the simulations; $Slab_{TZ}(t_{670})$ - the slab material in the transition zone at the time t_{670}; D_{TZ} - the slab accumulation rate in the transition zone; $Slab_{LM}$ (t_{670}) - the slab material in the lower mantle at the time t_{670}; D_{LM} - the slab accumulation rate in the lower mantle; *Classification* ($D_{TZ/LM}$) - ratio to classify the slab transition zone interaction (eq. 1) and convection style for a pair of young and old plate: 1 = both penetration/ whole mantle convection; 2 = both stagnant/ layered convection, T = old stagnant and young penetrating/ intermitted mantle convection.

Simulations	Plate age Myr	$(\gamma_{rw\text{-}pv};\ \gamma_{rw\text{-}pv+mw})$	ΔT_{pot}	Ra ($\times 10^{10}$)	$Rb_{rw\text{-}pv+mw}$ ($\times 10^{10}$)	$P_{rw\text{-}pv+mw}$	$\mathbf{t}_{670}$ Myr	$\mathbf{t}_{end}$ Myr	$Slab_{TZ}(t_{670})$ km^2	$Slab_{TZ\text{-}rate}$ km^2 Myr^{-1}	$Slab_{LM}(t_{670})$ km^2	$Slab_{LM\text{-}rate}$ km^2 Myr^{-1}	Classification
1	50	(3;-1)	-50	0.09	0.25	-0.04	35.9	121.76	47100	271	2830	2768	1
2	100			0.09	0.25	-0.04	21.08	106.67	48000	405	1440	4124	
3	50	(4;-2)		0.09	0.25	-0.07	43.42	115.83	45700	956	1770	1536	T
4	100			0.09	0.25	-0.07	17.64	139.68	37000	1940	1330	1867	
5	50	(5;-3)		0.09	0.25	-0.11	43.47	112.64	43700	1452	2400	996	2
6	100			0.09	0.25	-0.11	17.25	118.03	35400	2535	1770	1153	
7	50	(3;-1)	0	0.20	0.53	-0.04	13.17	81.33	30600	297	2050	3202	1
8	100			0.20	0.53	-0.04	10.59	64.45	39900	511	2470	5498	
9	50	(3.5;-1.5)		0.20	0.53	-0.05	13.06	84.32	30900	306	1680	3199	1
10	100			0.20	0.53	-0.05	10.16	67	40400	781	2100	5164	
11	50	(4;-2)		0.20	0.53	-0.07	12.74	83.07	31000	436	1590	3272	T
12	100			0.20	0.53	-0.07	10.01	112.56	41000	2976	2090	2327	
13	50	(4.5;-2.5)		0.20	0.53	-0.09	12.59	86.43	31400	572	1580	3122	T
14	100			0.20	0.53	-0.09	9.96	80.21	41100	2638	1870	1766	
15	50	(5;-3)		0.20	0.53	-0.11	12.52	92.24	31300	1127	1500	1956	T
16	100			0.20	0.53	-0.11	9.6	84.4	41300	2993	1890	1067	
17	50	(4;-2)	50	0.41	1.08	-0.07	7.07	86.63	27700	-4	0	4754	1
18	100			0.41	1.08	-0.07	5.71	68.53	36900	1290	969	6584	
19	50	(5;-3)		0.41	1.08	-0.11	6.5	111.77	27300	457	0	3022	1
20	100			0.41	1.08	-0.11	5.27	89.7	36800	570	443	6489	
20 (μ_{cut} 10^{23})	100			0.41	1.08	-0.04	5.22	85.38	36900	302	369	6955	N/A
20 (μ_{cut} 5×10^{22})	100			0.41	1.08	-0.04	5.19	85.2	37200	1781	221	4812	
20 (μ_{cut} 10^{22})	100			0.41	1.08	-0.07	4.96	70.69	37700	4817	0	2115	
21	50	(3;-1)	100	0.83	2.11	-0.07	5.55	25.61	28200	-91	1120	7397	1
22	100			0.83	2.11	-0.11	3.73	24.83	33000	93	618	9938	
23	50	(4;-2)		0.83	2.11	-0.11	5.1	35.86	29200	-296	166	8234	1
24	100			0.83	2.11	-0.04	3.47	38.84	33800	381	0	10970	
25	50	(5;-3)		0.83	2.11	-0.04	4.62	68.46	29100	-124	0	5263	1
26	100			0.83	2.11	-0.07	3.12	23.47	33500	2336	0	8664	
27	50	(3;-1)	200	3.02	7.11	-0.07	2.82	11.84	20600	-173	304	11258	1
28	100			3.02	7.11	-0.11	2.17	12.62	32900	-553	526	13280	
29	50	(4;-2)		3.02	7.11	-0.11	2.65	13.92	20600	-164	185	13280	1
30	100			3.02	7.11	-0.11	2.02	20.17	33200	411	28	13673	
31	50	(5;-3)		3.02	7.11	-0.11	2.41	28.37	19600	1788	37	5181	1
32	100			3.02	7.11	-0.11	1.77	21.5	32300	191	0	17539	

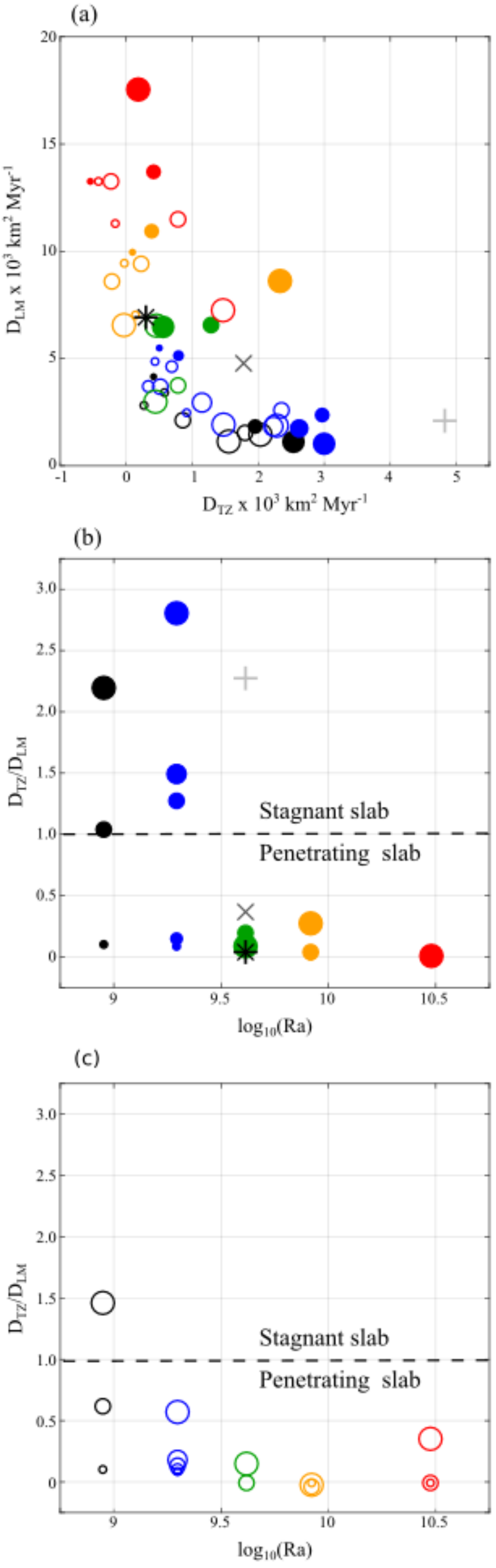

(a)
20
15
10
5
0
-1
0
1
2
3
4
5
D_LM x 10^3 km^2 Myr^-1
D_TZ x 10^3 km^2 Myr^-1
(b)
3.0
2.5
2.0
1.5
1.0
0.5
0
9
9.5
10
10.5
D_TZ/D_LM
Stagnant slab
Penetrating slab
log10(Ra)
(c)
3.0
2.5
2.0
1.5
1.0
0.5
0
9
9.5
10
10.5
D_TZ/D_LM
Stagnant slab
Penetrating slab
log10(Ra)

Supplementary Figure 1 – **Slab dynamics classification at different mantle temperatures**. (a) Slab accumulation rates in the transition zone (D_{TZ}) and in the lower mantle (D_{LM}) for all simulations. The open/solid symbols represent the models with the initial subducting plates of 50/100 Myr old, respectively. The symbol size and the colour represent the Clapeyron slopes, and mantle temperature respectively (see Figure 4). The asterisk, the cross and the plus symbols represents the additional cases with a cut off viscosity below 400 km reduced to 10^{23} Pa s, 5×10^{22} Pa s, and 10^{22} Pa s, respectively. (b,c) Accumulation ratio D_{TZ}/D_{LM} against Rayleigh number for 100-Myr (b) and 50-Myr (c) initial slab ages. Dashed horizontal black line at $D_{TZ}/D_{LM} = 1$ marks the regime boundary.

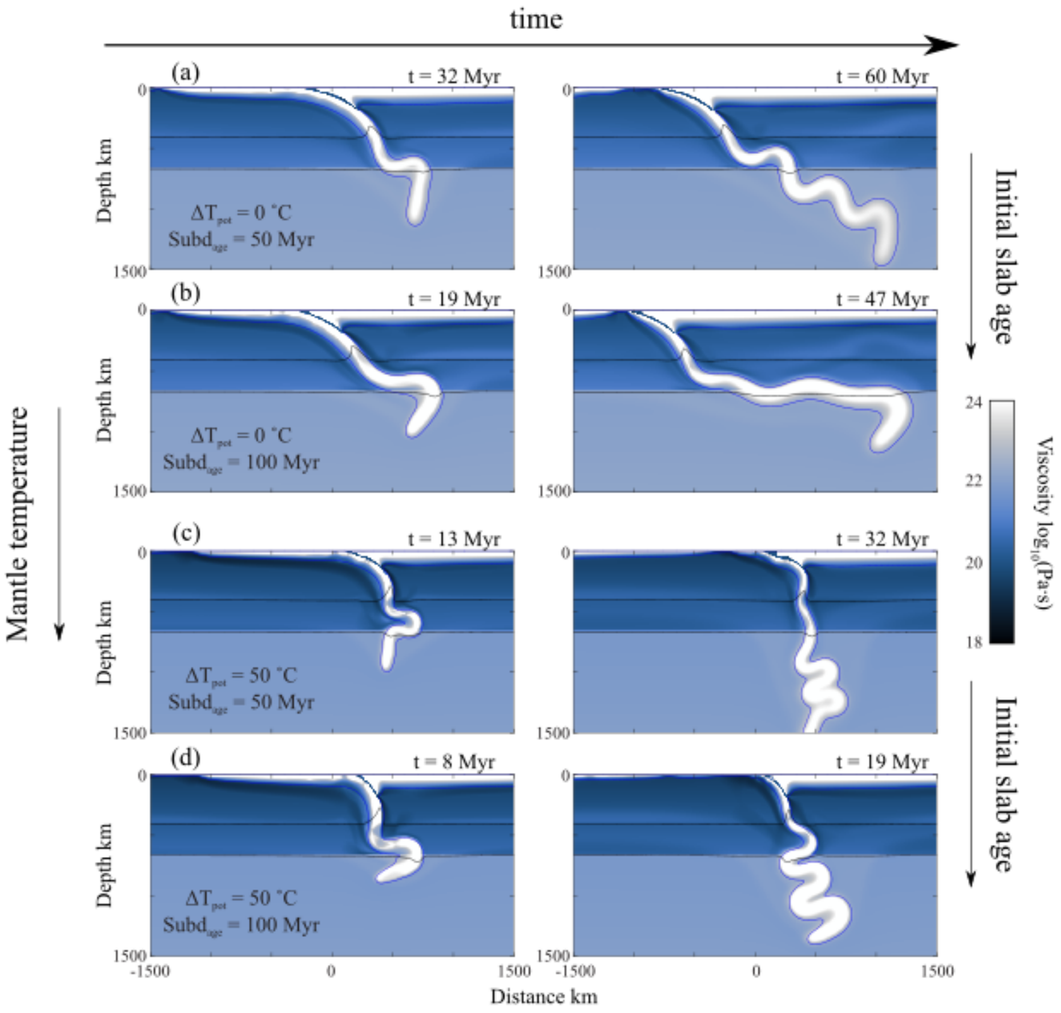

Supplementary Figure 2 – **Slab transition zone interaction in a mantle deforming with dislocation creep.** (a,b) Bi-modal slab-transition zone interaction at present-day temperature conditions for an endothermic phase transition of -2 MPa K^{-1} is illustrated by two snap shots each of the viscosity structure for the subduction of young (a) and old (b) plates. (c,d) Illustrate how in a model with the same parameters slab behaviour changes at higher mantle temperature (ΔT_{pot} = 50 °C) (c, young and d, old plate). In these cases both plates sink readily into the lower mantle. These results agree with the model when only a Newtonian rheology is considered.

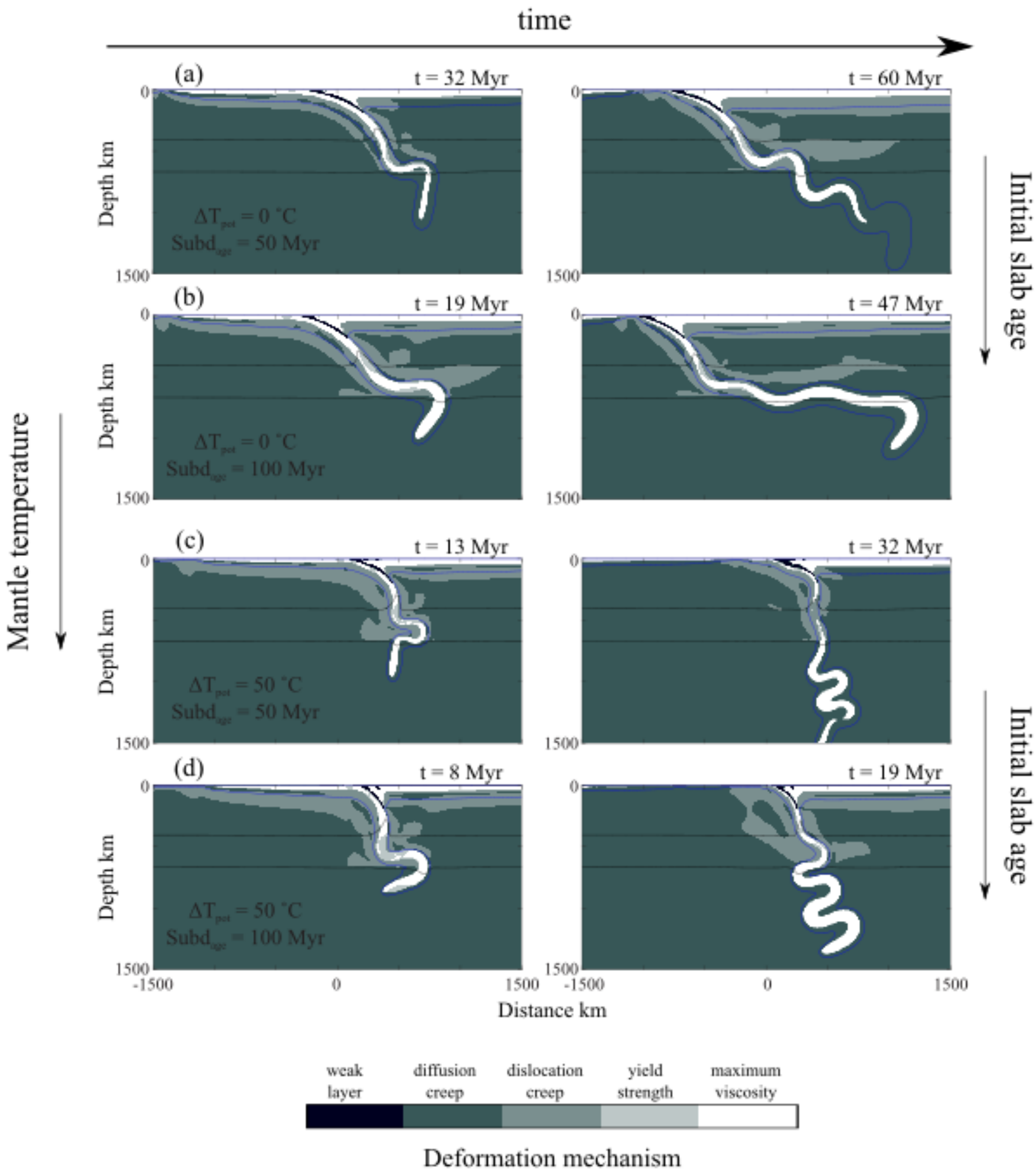

Supplementary Figure 3 – **Dominant deformation mechanism during slab sinking for non-Newtonian models.** Same snapshots as Supplementary Fig 2, but illustrating the dominant (weaker) deformation mechanism. Dislocation creep is limited to the asthenosphere and around the slab where stresses are highest. (a,b) Bi-modal slab-transition zone interaction at present-day temperature conditions for an endothermic phase transition of -2 MPa K^{-1} for the subduction of young (a) and old (b) plates. (c,d) Model with the same parameters of (a) and (b) but at higher mantle temperature (ΔT_{pot} = 50 °C) (c, young and d, old plate). In these cases both plates sink readily into the lower mantle.
